# Some taxonomic notes on the genus *Oberea* Dejean, 1835 from Asia (Coleoptera, Cerambycidae, Lamiinae)

**DOI:** 10.3897/zookeys.647.11120

**Published:** 2017-01-27

**Authors:** Zhu Li, Giulio Cuccodoro, Li Chen

**Affiliations:** 1 College of Plant Protection, Southwest University, Chongqing 400715, China; 2 Muséum d’histoire naturelle, CH-1211 Genève 6, Switzerland

**Keywords:** New country record, new synonym, Oberea, restoration name, taxonomy

## Abstract

In the current work, the following taxonomic changes of genus *Oberea* Dejean, 1835 are proposed: *Oberea flavescens* Breuning, 1947, **rest. stat.**; *Oberea toi* Gressitt, 1939, **rest. stat.**; *Oberea sylvia* Pascoe, 1858, **rest. stat.**; *Oberea taiwana* Matsushita, 1933 = *Oberea taihokuensis* Breuning, 1962, **syn. n.**; *Oberea sumbana* Breuning, 1961 = *Oberea antennata* Franz, 1972, **syn. n.**; *Oberea brevithorax* Gressitt, 1939 is newly recorded from Vietnam.

## Introduction

The genus *Oberea* Dejean, 1835, belonging to the subfamily Lamiinae, family Cerambycidae, consists of more than 300 species worldwide. The two monographic books on the taxonomy of Cerambycidae from China (Gressitt 1951) and Laos (Rondon and Breuning 1970), the most important works on *Oberea* in Asia, treated 77 and 20 *Oberea* species, respectively. *Oberea* worldwide was revised by Breuning (1960–1962). Some species were downgraded to infrasubspecific rank in Breuning’s work basing on the similarity of body coloration (Breuning 1960-1962), and some of them were restored or described as new species (Kurihara and Ohbayashi 2007; Kurihara 2009; Li et al. 2014, 2016). During the recent study, some further taxonomic clarification of the genus *Oberea* is presented in the current work, based on examination of types.

## Material and methods

Pictures of adult morphology are composites taken using a digital camera mounted onto a Leica MZ Apo dissecting microscope and subsequently processed using Automontage® software. For detailed examination, genitalia were extracted from specimens softened in water, cleared in 10% KOH, observed in water on glass microscope slides, then transferred into ethanol 70% and stored in capsules mounted on the same pin as the specimens. Drawings were made using a drawing tube mounted onto a compound microscope.

The following collection abbreviations are used in the text.

BMNH
The Natural History Museum, London, UK


MNHN
Muséum national d’Histoire Naturelle, Paris, France


MHNG
Muséum d’histoire naturelle, Geneva, Switzerland


MHNL
Musée des Confluences, Lyon, France


NMB
Musée d’Histoire Naturelle de Bâle, Basel, Switzerland


SWU
Insect Collection of Southwest University, Chongqing, China


SYSU
Sun Yat-sen University (ex Lingnan National History Museum or Zhongshan University), Guangzhou, China


## Taxonomy

### Oberea flavescens

Taxon classificationAnimaliaColeopteraCerambycidae

Breuning, 1947 rest. stat.

Figs 1 , 2

Oberea flavescens Breuning, 1947: 146. Type locality: China, Sichuan.
Oberea atropunctata v. flavescens : Breuning 1962: 169.

#### Redescription.

**Figure 1.**
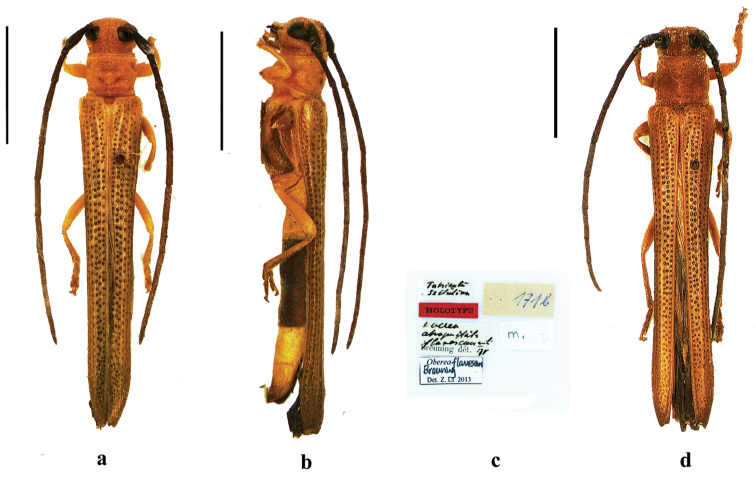
Habitus of *Oberea flavescens* Breuning, 1947, **a–c** holotype, female, from Sichuan **a** dorsal view **b** lateral view **c** label (not to scale) **d** male, from Sichuan, dorsal view. Scale bar 5.0 mm.

*Body* (Fig. 1) 17.5–17.9 mm long and 3.0 mm wide. Head ochreous, apical mandible dark brown; antennae reddish brown, scape dark brown; ventral surface (except the abdominal sternite II and III black) and legs pale yellowish brown, tarsi darker. Body clothed with short golden pubescence and some erect hairs on pronotum, base of elytra and ventral surface of the basal antennal segments. *Head* slightly narrower than prothorax, vertex distinctly depressed at middle with a groove; eyes very large, inferior lobes twice as long as the gena in male and 1.5 times in female. Antennae distinctively shorter than body, reaching the apical fourth of elytra, antennomere ratio: 12.3: 2.5: 16.0: 15.8: 15.8: 15.9: 15.7: 15.4: 13.5: 12.6: 12.5 in males and 14.1: 3.0: 14.8: 14.2: 14.1: 14.9: 15.3: 15.1: 14.8: 14.4: 13.6: 13.5 in females. *Prothorax* wider than long; apical and basal margins slightly emarginated; sides rounded at middle, slightly constricted basally and apically; pronotum raised in middle, finely and densely punctured. *Scutellum* squared, slightly emarginated. *Elytra* very long, nearly 5.5 times as long a humeral width, and 4.5 times as long as head and prothorax combined, slightly narrowed from behind base to apical quarter, basal punctured arranged in 6 longitudinal series, and the punctures large and deep at basal area, gradually finer and irregular towards apex. *Metepisternum* and sides of abdominal segments finely punctured. Hind femora reaching posterior edge of abdominal segment I; hind tibiae almost twice as long as tarsi. Abdominal sternite V with a shallow triangular concave in males and with a median longitudinal groove in females.

**Figure 2.**
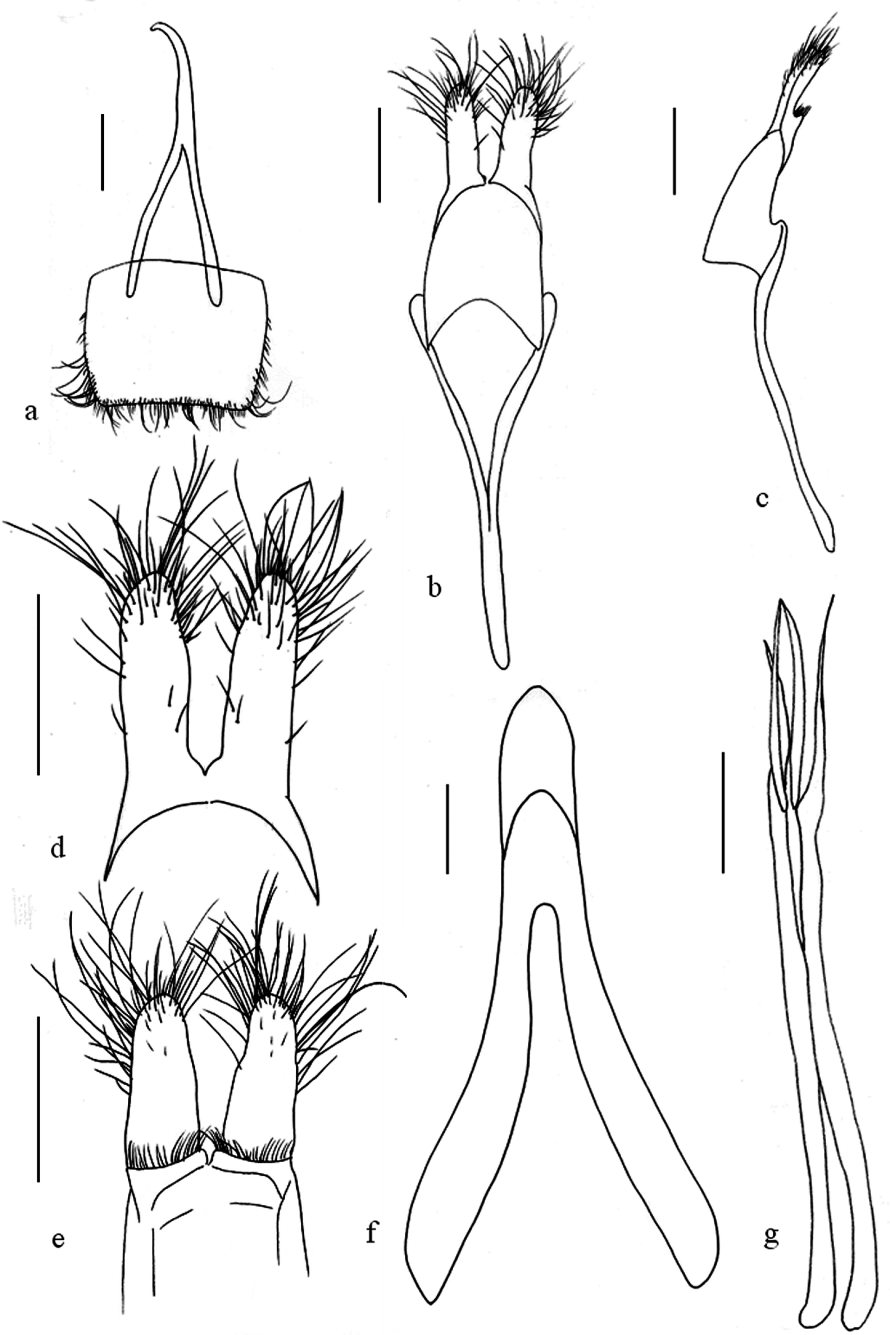
*Oberea flavescens*, male genitalia, **a** tergite viii **b** tegmen, dorsal view **c** tegmen, lateral view **d** parameres, dorsal view **e** parameres, ventral view **f** median lobe, ventral view **g** sclerities in endophallus. Scale bar 0.5 mm.

*Male terminalia*: (Fig. 2) Tergite VIII broader than long, apex truncated and slightly emarginated, densely clothed with short setae (Fig. 2a); tegmen curved in profile, parameres elongate, mostly covered with long setae at the apical half; base of each parameres transversely and obliquely ridged on ventral side; the ridge covered with dense fine hairs (Fig. 2b-e); Median lobe 1.1 times as long as tegmen and slightly curved in profile; the median struts 3/5 times as long as the whole median lobe in length; dorsal plate slightly longer than ventral plate; apex of ventral plate rounded; median foramen rounded (Fig. 2f); endophallus with 2 pairs of rods at apical portion; longer pair very slender baculiform, about 3.5 times as long as shorter pair (Fig. 2g).

**Figure 3.**
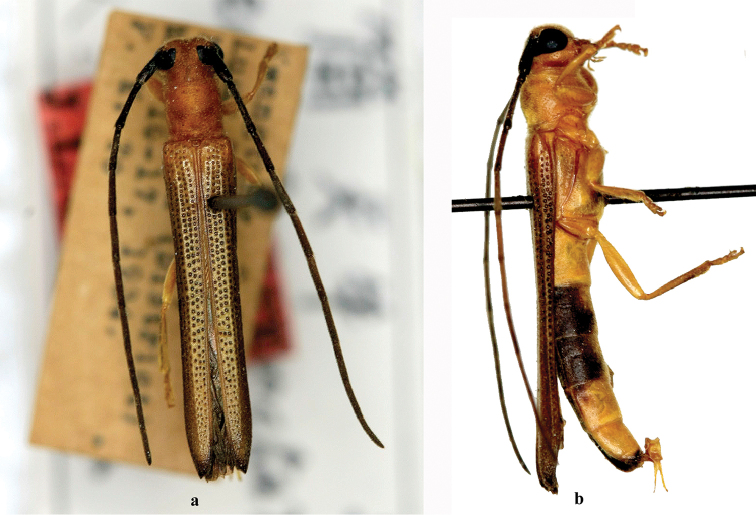
Habitus of *Oberea toi* Gressitt, 1939, holotype, female, from Guangdong, **a** dorsal view **b** lateral view. (not to scale).

#### Type material examined.

*Oberea flavescens* Breuning: Holotype, ♀, Chine, Szetschuan, Tatsienlu (MNHG).

#### Additional material examined.

**China**, Sichuan: 1♂, Chine, Szetschuan, Tatsienlu (MHNG) [a mislabeled paratype]

#### Distribution.

China (Sichuan).

#### Remarks.

**Figure 4.**
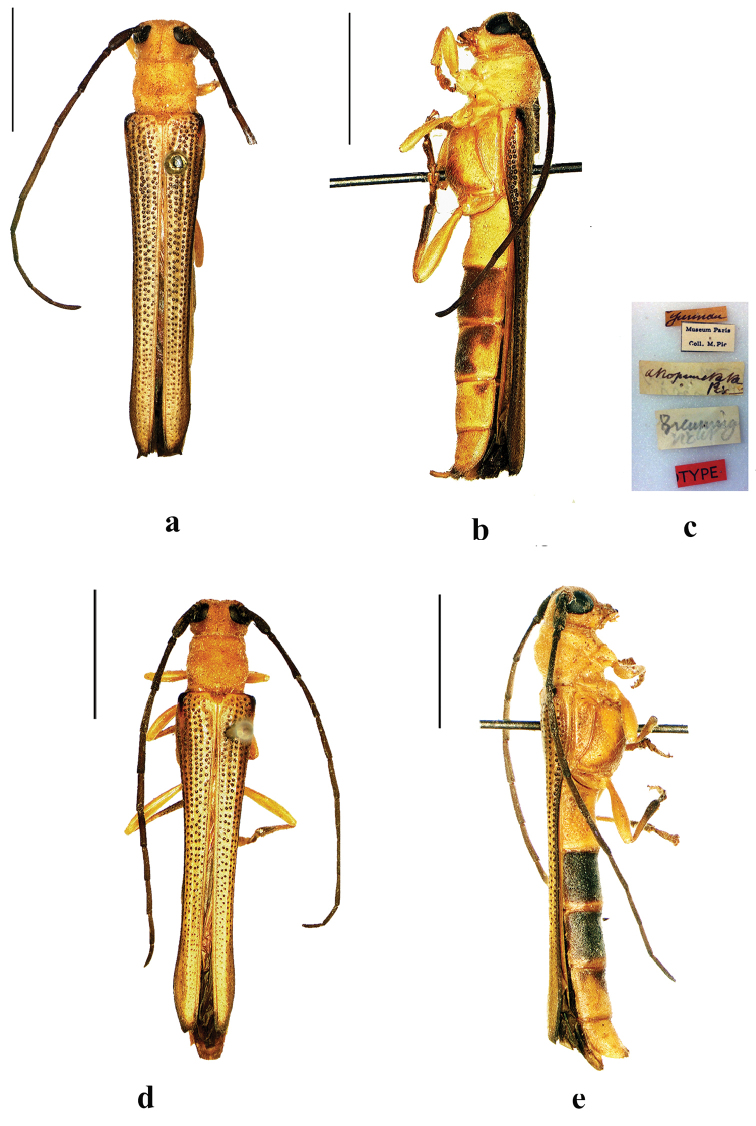
Habitus of *Oberea atropunctata* Pic, 1916, **a–c** holotype, female, from Yunnan **a** dorsal view **b** lateral view **c** label (not to scale) **d–e** male, from Yunnan **d** dorsal view, **e** lateral view. Scale bar 5.0 mm.

**Figure 5.**
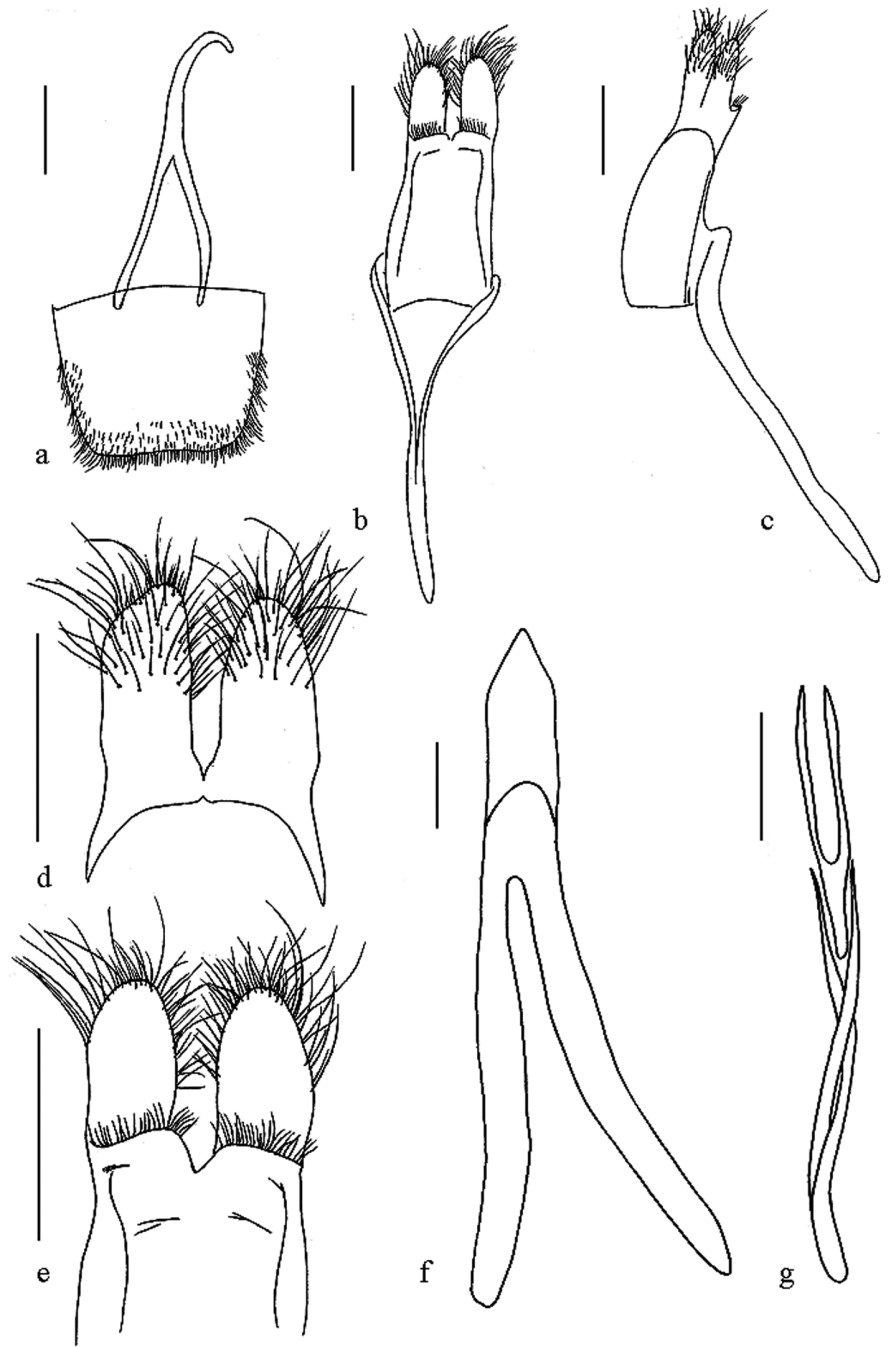
*Oberea atropunctata*, male genitalia, **a** tergite viii **b** tegmen, dorsal view **c** tegmen, lateral view **d** parameres, dorsal view, **e** parameres, ventral view **f** median lobe, ventral view **g** sclerities in endophallus. Scale bar 0.5 mm.

This species was first described by Breuning in 1947 based on a specimen from Sichuan Province, China but it was downgraded to a variety of *Oberea atropunctata* Pic, 1916 in his revisionary work (1960–1962). A careful examination and comparison of the types of *Oberea flavescens* and *Oberea atropunctata* (Figs 4–5) show that they are different species. *Oberea flavescens* differs from *Oberea atropunctata* in having longer elytra and shorter antennae. They can be distinguished by having differently shaped male genitalia, the long pair of rods being 3.5 times as long as the short pair (1.5 times as long as in *Oberea atropunctata*) and the short pair consisting of two simple short rods.

### Oberea toi

Taxon classificationAnimaliaColeopteraCerambycidae

Gressitt, 1939 rest. stat.

Fig. 3

Oberea toi Gressitt, 1939a: 106. Type locality: China, Guangdong.
Oberea atropunctata v. toi : Breuning 1962: 170.

#### Type material examined.

*Oberea toi* Gressitt: Holotype, ♀, Lung-ping-hui, Lien District, N. Kwangtung Prov., 16. V. 1934, F. K. To Coll. (SYSU).

#### Additional material examined.

**China**, Jiangxi: 1♀, Kiukiang (MNHG).

#### Distribution.

China (Guangdong, Jiangxi).

#### Remarks.

*Oberea toi* Gressitt, 1939 was originally described by Gressitt from Guangdong, China, and then was downgraded to a variety of *Oberea atropunctata* by Breuning (1960–1962). It was regarded as the synonym of *Oberea atropunctata* (Löbl & Smetana, 2010); however, the examination of the types shows that they are different species. Despite the similar color pattern of their bodies, *Oberea toi* differs from *Oberea atropunctata* in the following characters: antennae as long as the body in female, and hind femora exceeding the posterior edge of abdominal segment I. Therefore, we suggest restoring *Oberea toi* Gressitt, 1939 from synonymy of *Oberea atropunctata* Pic, 1916.

### Oberea atropunctata

Taxon classificationAnimaliaColeopteraCerambycidae

Pic, 1916

Figs 4 , 5

Oberea atropunctata Pic, 1916: 17. Type locality: China, Yunnan.

#### Description.

*Male terminalia*: (Fig. 5) Tergite VIII broader than long, apex truncated and slightly emarginated, rounded at side, densely clothed with short setae (Fig. 5a); tegmen curved in profile, parameres stouter, mostly covered with long hairs; transverse and oblique ridge at basal lobe on ventral side with dense fine hairs (Fig. 5b–e); penis 1.25 times as long as tegmen and curved in profile; the median struts 4/5 times as long as the whole median lobe in length; dorsal plate slightly longer than ventral plate; apex of ventral plate rounded; median foramen rounded (Fig. 5f); endophallus with two pairs of sclerites apically, the long pair very slender baculiform, the short pair fused at base forming a “Y” (Fig. 5g).

#### Diagnosis.

Body 17.9 mm long and 3.0 mm wide. The species is very similar to *Oberea flavescens* Breuning and *Oberea toi* Gressitt, especially in color patterns. The following combination of characters separates it from *Oberea flavescens*: shape of rods in the endophallus; elytra nearly 4.2 times as long as humeral width, and 3.7 times as long as head and prothorax combined.

#### Type material examined.

*Oberea atropunctata* Pic: Holotype, ♀, Yunnan (MNHN).

#### Additional material examined.

**China**, Sichuan: 1♂, Sichuan Province, 8.V.1985 (SWU); Yunnan: 1♂, China, Yun-nan-sen (MNHG).

#### Distribution.

China (Sichuan, Yunnan).

### Oberea taiwana

Taxon classificationAnimaliaColeopteraCerambycidae

Matsushita, 1933

Fig. 6

Oberea taiwana Matsushita, 1933: 423. Type locality: China, Taiwan.
Oberea taihokuensis Breuning, 1962: 168. Type locality: China, Taiwan. **syn. n.**
Oberea taihokuensis v. *flavosternalis* Breuning, 1962: 169. [Unavailable name according to ICZN, art. 10.2.]

#### Type material examined.

*Oberea taihokuensis* Breuning: Holotype, ♀, Formosa, Taihoku, 20.IV.1932, coll. M. Chujo (MHNG).

#### Additional material examined.

**China**, Taiwan: 1♂, Formosa, Kosempo (MHNG) [holotype of *Oberea taihokuensis* v. *flavosternalis*]; 1♀, Formosa, Kurau [a mislabeled Paratype].

#### Distribution.

China (Taiwan).

#### Remarks.

Matsushita described *Oberea taiwana* from Taiwan, China in 1933. In Breuning’s revision (1960–1962) on worldwide *Oberea*, *Oberea taiwana* Matsushita was recorded without examining types and *Oberea taihokuensis* was described as a new species in 1962. Kurihara and Ohbayashi (2007) revised the *Oberea* species from Taiwan and re-described *Oberea taihokuensis* based on the original description. According to the original description of *Oberea taiwana*, the type was deposited in Hokkaido University, but Dr. Kurihara could not find any type there (Kurihara *in litt*.). After having compared photo and description of *Oberea taiwana* in Kurihara and Ohbayashi’s publication (2007) and the holotype of *Oberea taihokuensis*, it is suggested that *Oberea taihokuensis* Breuning, 1962 is junior synonym of *Oberea taiwana* Matsushita, 1933.

**Figure 6.**
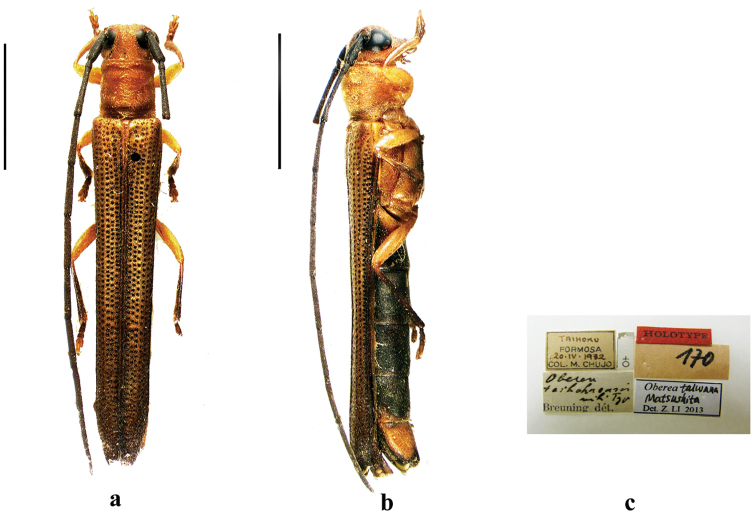
Habitus of *Oberea taihokuensis* Breuning, 1962, holotype, female, from Taiwan, **a** dorsal view **b** lateral view, **c** label (not to scale). Scale bar 5.0 mm.

### Oberea sylvia

Taxon classificationAnimaliaColeopteraCerambycidae

Pascoe, 1858 rest. stat.

Figs 7 , 8

Oberea sylvia Pascoe, 1858: 261. Type locality: “China, Borealis”.

#### Redescription

**Figure 7.**
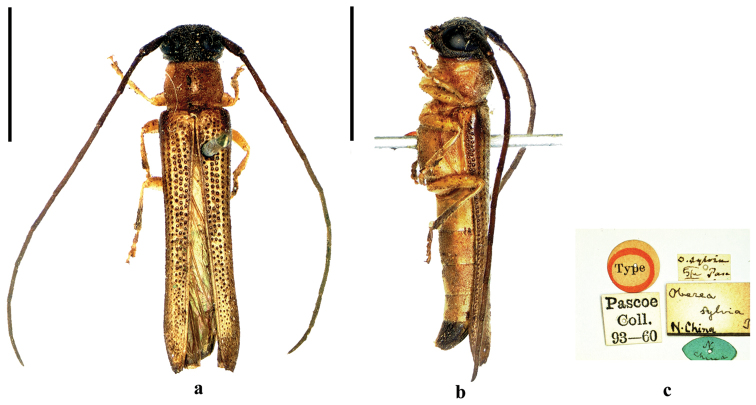
Habitus of *Oberea sylvia* Pascoe, 1958, holotype, male, from North China, **a** dorsal view **b** lateral view **c** label (not to scale). Scale bar 5.0 mm.

**(Fig. 7).** *Body* 12.5–13.5 mm long and 2.3mm wide. *Head* black except for labrum yellowish brown to reddish brown, maxillary palpus and labial palpus pale yellowish brown; antennae reddish brown, scape dark brown. *Prothorax*, elytra and ventral surface (except for the abdominal segment V black) ochraceous; legs yellowish brown, apical half of hind tibia and tarsi darker brown. Body clothed with short golden pubescence and some erect hairs on pronotum, base of elytra and ventral surface of the basal antennal segments. *Head* short, with distinctly depressed vertex; eyes very large, inferior lobes 2 times as long as the gena in male. Antennae of males longer than body, antennomere III longer than pedicel and antennomere IV. *Prothorax* 1.2 times wider than long, slightly constricted basally and apically; pronotum with a tubercle in middle, finely and densely punctured. Scutellum squared, slightly emarginated. Elytra nearly three times as long as humeral width, and 3.6 times as long as head and prothorax combined, slightly narrowed from behind base to apical quarter, apex truncate; basal disc with large and deep punctures arranged in line, punctures becoming gradually finer and irregular towards apical quarter. *Metepisternum* and sides of abdominal surface finely punctate. Metafemora reaching posterior edge of abdominal segment I; metatibiae almost twice as long as tarsi. Abdominal sternite V with a shallow triangular concave in males.

**Figure 8.**
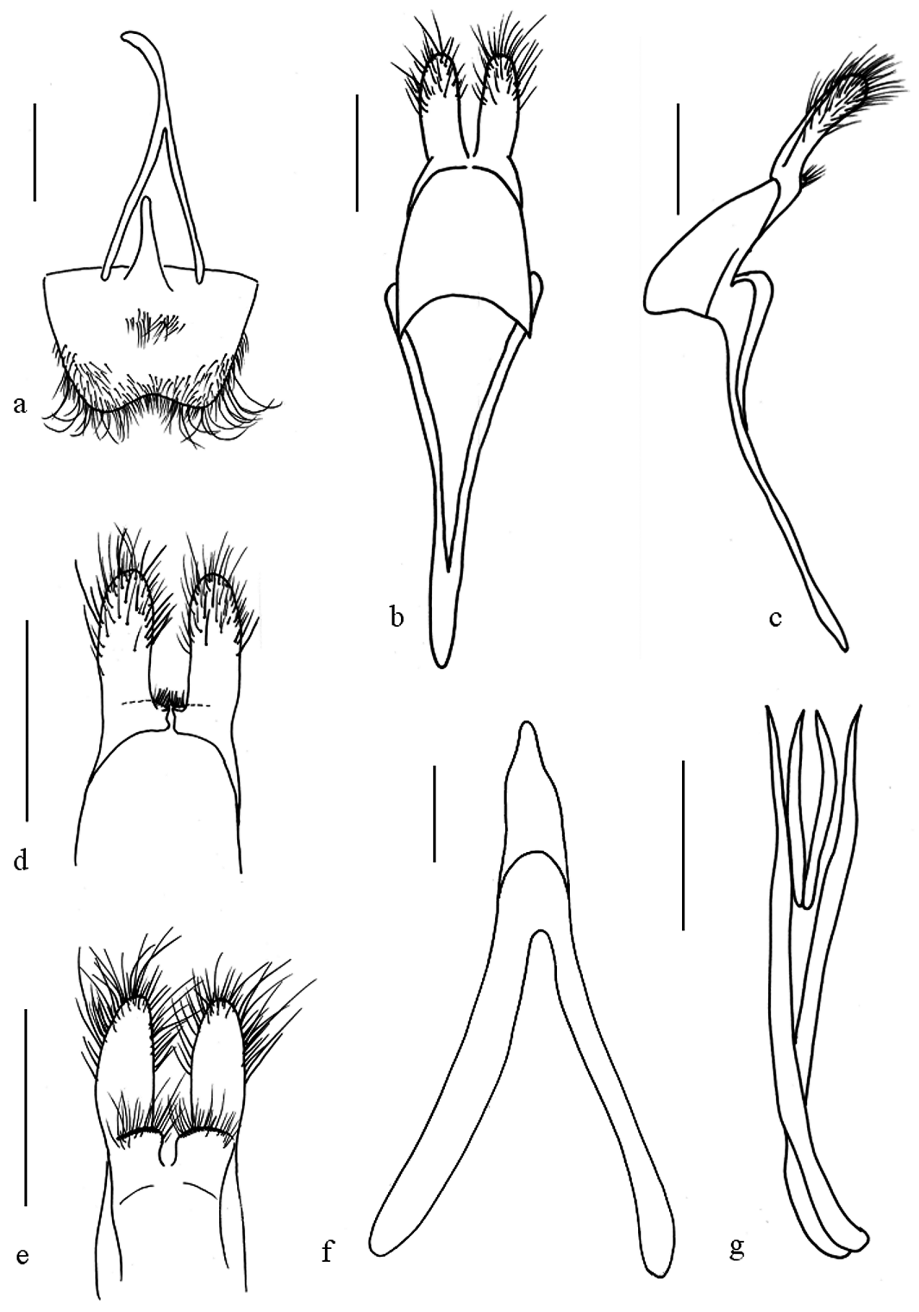
*Oberea sylvia*, male genitalia, **a** tergite viii **b** tegmen, dorsal view **c** tegmen, lateral view **d** parameres, dorsal view **e** parameres, ventral view **f** median lobe, ventral view, **g** sclerities in endophallus. Scale bar 0.5 mm.

*Male terminalia*. (Fig. 8) Tergite VIII broader than long, apex truncated and slightly emarginated, rounded at sides, densely clothed with long hairs and short setae (Fig. 8a); Tegmen curved and penis curved in profile, parameres mostly covered with long setae on the apical half; base of each lobe in ventral side transversely and obliquely ridged; the ridge with dense fine hairs (Fig. 8b–e); penis 1.2 times as long as tegmen, dorsal plate slightly longer than ventral plate; the median struts 3/5 times as long as the whole median lobe in length; apex of the ventral plate rounded; median foramen rounded (Fig. 8f); apical endophallus with 2 pairs of baculiform rods, the long pair 2.8 times as long as short pair (Fig. 8g).

#### Type material examined.

*Oberea sylvia* Pascoe: Holotype, ♂, N. China (BMNH).

#### Additional material examined.

**China**: 1♂, Chine (MHNL); 4♂♂, Chine (BMNH).

#### Distribution.

East China.

#### Remarks.

*Oberea sylvia* was originally described by Pascoe 1858 and regarded as synonym of *Oberea nigriceps* (White, 1844) (Breuning, 1962). After comparing the types, *Oberea sylvia*, which distinctly differs from *Oberea nigriceps* in male genitalia (Fig. 8), is restored to specific rank.

The holotype probably was collected by Robert Fortune. According to his book “Three years wandering in the north provinces of China”, the northern province of China included Shanghai, Zhejiang Province and Jiangsu Province; therefore “N. China” or “China borealis” might mean east China. Unfortunately, there is no detailed information about the location of the specimen that the first author examined.

### Oberea brevithorax

Taxon classificationAnimaliaColeopteraCerambycidae

Gressitt, 1936

Fig. 9

Oberea brevithorax Gressitt, 1936: 108. Type locality: China, Taiwan.
Oberea brevithorax inepta Gressitt, 1939b: 122. Type locality: China, Fujian.
Oberea binotaticollis v. brevithorax : Breuning 1962: 193.
Oberea binotaticollis v. inepta : Breuning 1962: 193.
Oberea brevithorax : Kurihara and Ohbayashi 2007: 211.

#### Type material examined.

*Oberea brevithorax inepta* Gressitt: Holotype, ♂, Cha Shan, Kien-ning District, Fukien Province, SE. China, VI. 22-28. 1933. D. C. Ngu coll. (SYSU).

#### Additional material examined.

**China**, Zhejiang: 1♂, Zhejiang province, Lin’an city, West Tianmushan, Dajingwu, 30°22'18.86"N,119°26'03.81"E, 828m, 9–11. VII.2012, leg. Jianyue Qiu and Hao Xu (SWU); **Vietnam**, 1♂, Chapa, Tonkin, J. Clemont coll. (MHNG); 1 ♂, Chapa, Tonkin (MHNG).

#### Distribution.

China (Fujian, Hainan, Zhejiang, Taiwan) ; Vietnam (new record).

#### Remarks.

*Oberea brevithorax* was first described as a valid species by Gressitt in 1936 but was downgraded as a variety of *Oberea binotaticollis* Pic, 1915 by Breuning in his revision. Kurihara and Ohbayashi (2007) compared them and confirmed that they were two different species, easily distinguished from each other by different body proportions, antennal lengths, and male genitalia. The species was only recorded in China but recently, some specimens collected in Tonkin, Vietnam, were found in MHNG, and they are a new record to Vietnam.

**Figure 9.**
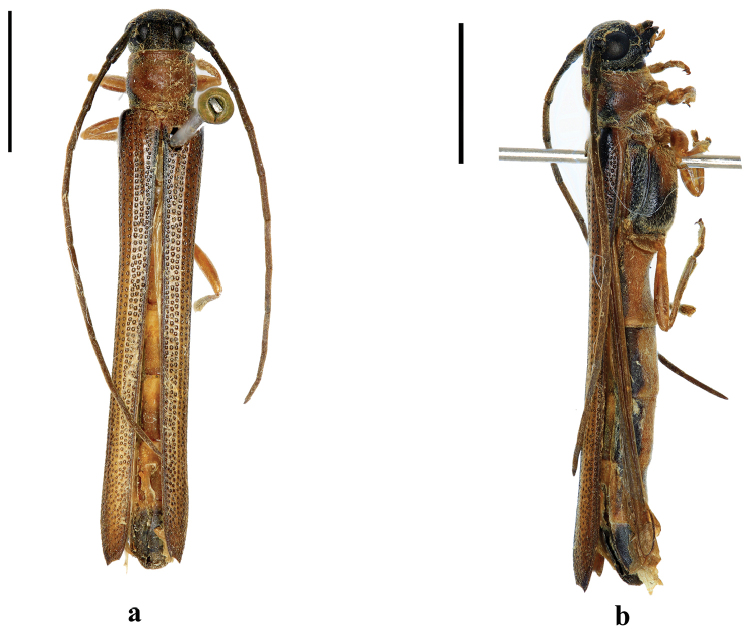
Habitus of *Oberea brevithorax* Gressitt, 1936, male, from Tonkin, **a** dorsal view **b** lateral view. Scale bar 5.0 mm.

### Oberea sumbana

Taxon classificationAnimaliaColeopteraCerambycidae

Breuning, 1961

Figs 10 , 11

Oberea sumbana Breuning, 1961: 131.Type locality: Indonesia, Sumba.
Oberea antennata Franz, 1972: 143. Type locality: Indonesia, Sumba. **syn. n.**

#### Type material examined.

*Oberea sumbana* Breuning: Holotype, ♂, Sumba (MHNG); Allotype: ♀, Waingapoe, 96, [P.] Everett [printed label faded] (MHNG). *Oberea antennata* Franz: Holotype, ♂, O. Sumba: Melolo Iaewa, 28.V.1949, Dr. Bühler & Dr. Sutter leg. (NMB); Allotype: ♀, C. Sumba: Langgaliru, 6.10.1949. Dr. Bühler & Dr. Sutter leg. (NMB).

#### Distribution.

Indonesia.

#### Remarks.

After examining the holotypes of *Oberea sumbana* and *Oberea antennata*, it is concluded that *Oberea antennata* Franz, 1972 is junior synonym of *Oberea sumbana* Breuning, 1961.

**Figure 10.**
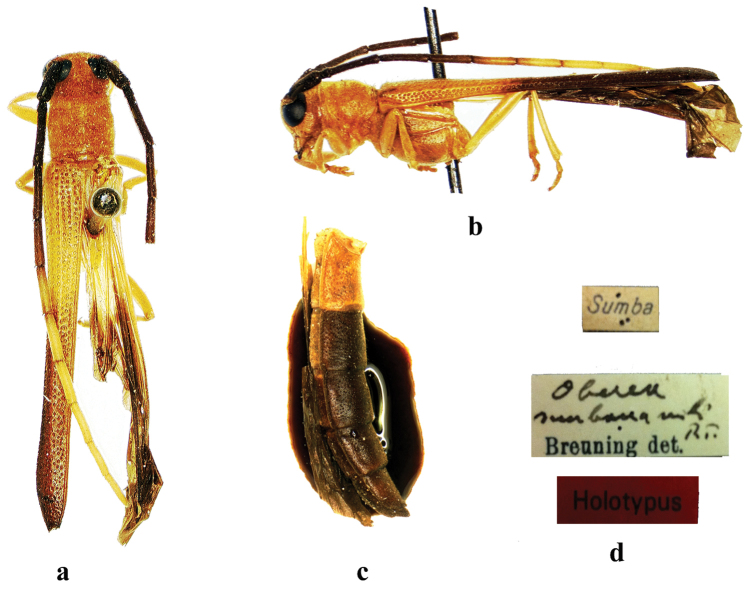
Habitus of *Oberea sumbana* Breuning, 1961, holotype, male, from Sumba, **a** dorsal view **b** lateral view (without abdomen) **c** abdomen, lateral view **d** label. (not to scale).

**Figure 11.**
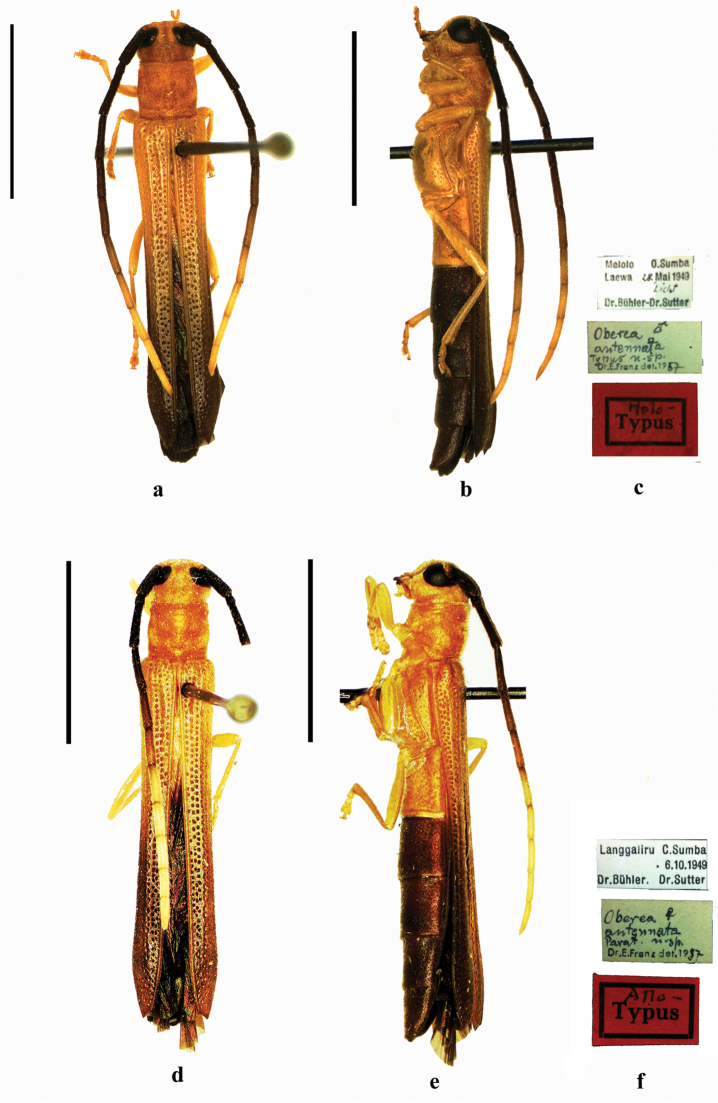
Habitus of *Oberea antennata* Franz, 1972, **a–c** holotype, male, from Sumba **a** dorsal view **b** lateral view, **c** label (not to scale) **d–f** allotype, female, from Sumba **d** dorsal view **e** lateral view **f** label (not to scale). Scale bar 5.0 mm.

## Supplementary Material

XML Treatment for Oberea flavescens

XML Treatment for Oberea toi

XML Treatment for Oberea atropunctata

XML Treatment for Oberea taiwana

XML Treatment for Oberea sylvia

XML Treatment for Oberea brevithorax

XML Treatment for Oberea sumbana
